# Construction of High-Resolution RAD-Seq Based Linkage Map, Anchoring Reference Genome, and QTL Mapping of the Sex Chromosome in the Marine Medaka *Oryzias melastigma*

**DOI:** 10.1534/g3.119.400708

**Published:** 2019-09-17

**Authors:** Bo-Young Lee, Min-Sub Kim, Beom-Soon Choi, Atsushi J. Nagano, Doris Wai Ting Au, Rudolf Shiu Sun Wu, Yusuke Takehana, Jae-Seong Lee

**Affiliations:** * Department of Biological Science, College of Science, Sungkyunkwan University, Suwon 16419, South Korea,; † Phyzen Genomics Institute, Seongnam 13558, South Korea,; ‡ Faculty of Agriculture, Ryukoku University, Kyoto 612-8577, Japan,; § Department of Chemistry and; ** State Key Laboratory of Marine Pollution, The City University of Hong Kong, Hong Kong SAR, China,; †† Department of Science and Environmental Studies, The Education University of Hong Kong, Hong Kong SAR, China, and; ‡‡ Department of Animal Bioscience, Nagahama Institute of Bio-Science and Technology, Nagahama, Shiga 526-0829, Japan

**Keywords:** marine medaka, *Oryzias melastigma*, linkage map, reference genome, sex, quantitative trait locus

## Abstract

Medaka (*Oryzias* sp.) is an important fish species in ecotoxicology and considered as a model species due to its biological features including small body size and short generation time. Since Japanese medaka *Oryzias latipes* is a freshwater species with access to an excellent genome resource, the marine medaka *Oryzias melastigma* is also applicable for the marine ecotoxicology. In genome era, a high-density genetic linkage map is a very useful resource in genomic research, providing a means for comparative genomic analysis and verification of *de novo* genome assembly. In this study, we developed a high-density genetic linkage map for *O. melastigma* using restriction-site associated DNA sequencing (RAD-seq). The genetic map consisted of 24 linkage groups with 2,481 single nucleotide polymorphism (SNP) markers. The total map length was 1,784 cM with an average marker space of 0.72 cM. The genetic map was integrated with the reference-assisted chromosome assembly (RACA) of *O. melastigma*, which anchored 90.7% of the assembled sequence onto the linkage map. The values of complete Benchmarking Universal Single-Copy Orthologs were similar to RACA assembly but N50 (23.74 Mb; total genome length 779.4 Mb; gap 5.29%) increased to 29.99 Mb (total genome length 778.7 Mb; gap 5.2%). Using MapQTL analysis with SNP markers, we identified a major quantitative trait locus for sex traits on the Om10. The integration of the genetic map with the reference genome of marine medaka will serve as a good resource for studies in molecular toxicology, genomics, CRISPR/Cas9, and epigenetics.

Many fish species are useful for ecotoxicological research, as they indicate an early warning of environmental contamination caused by various aquatic pollutants (Arellano-Aguilar *et al.* 2009). Recently, an increase in the contamination levels of estuaries and coastal water due to anthropogenic pollutants emphasizes the need for marine sentinel model fish species. Medaka (*Oryzias* sp.) is an important fish species in ecotoxicology and considered a model species, as its biological features include certain advantages such as small body size and short generation time (Kim *et al.* 2016). Japanese medaka *Oryzias latipes* is important and widely used model species for the studies of genetics, evolution, and ecotoxicology, with abundant genomic resources. Since *O. latipes* is a freshwater species, their responses to environmental toxicants can be different in those of marine fish (Shi *et al.* 2008; Wu *et al.* 2012; Wheeler *et al.* 2002). Marine medaka *Oryzias melastigma* inhabits brackish water in Asian regions including Pakistan, India, Burma, and Thailand (Naruse 1996). *O. melastigma* has been acknowledged as a potential model fish for marine ecotoxicological studies and is useful for the evaluation of acute and/or chronic toxicity, and embryo toxicity testing (Chen *et al.* 2011; Dong *et al.* 2014; Kim *et al.* 2016; Kong *et al.* 2008; Shen *et al.* 2010; Wang *et al.* 2011; Kim *et al.* 2016). The genus *Oryzias* has been divided into three major monophyletic species groups; the *latipes*, the *javanicus*, and the *celebenesis* groups, while *O. dancena*, a closely related species of *O. melastigma*, has been phylogenetically placed in the *javanicus* group (Takehana *et al.* 2005). Phenotypic distinction between male and female in medaka is distinguished by a number of secondary sex characters including shape and size of dorsal and anal fins due to morphologically indistinguishable sex chromosomes (Schartl 2004). Moreover, *Oryzias* species shows the both XY and ZW sex-determining systems. *O. latipes*, *O. dancena*, and *O. minutillus* have an XX/XY sex-determining system and *O. hubbsi* has a ZZ/ZW (Matsuda *et al.* 1998; Takehana *et al.* 2008). For example, sex-determining gene *dmrt1bY* has been identified in a few species of the *O. latipes* group and *sox3^Y^* in *O. dancena* and some species of the *celebenesis* group (Kondo *et al.* 2003; Matsuda *et al.* 2002; Myosho *et al.* 2015). There is a lack of studies on sex-determining genes in *O. melastigma*. Therefore, more genomic resources specifically devoted for the marine medaka *O. melastigma* are required.

A genetic linkage map is a very useful tool to understand genetic architecture such as chromosome structure, segregation distortion regions, recombination rate, and recombination hotspots (Meyer and Van de Peer 2005; Zhu *et al.* 2014; Guo *et al.* 2013; Shifman *et al.* 2006). Furthermore, it provides a framework for mapping the chromosomal location of single-gene traits and quantitative traits of interest, and helps to facilitate candidate gene cloning, and comparative genomic analysis with some genome information together (Lee *et al.* 2005; Amores *et al.* 2011; Feng *et al.* 2015; Zhu *et al.* 2013; Li *et al.* 2015; Shao *et al.* 2015; Wang *et al.* 2015; Xiao *et al.* 2015; Kanamori *et al.* 2016). A high-density genetic map also plays an important role in assembling whole genome sequences by examining the accuracy of *de novo* genome assembly (Dukić *et al.* 2016; Rhee *et al.* 2017; Wang *et al.* 2017). Indeed, the importance of a high-density genetic map has been demonstrated during the *de novo* genome assembly in teleost fish, as it validated the presence of additional genome duplication (Meyer and Schartl 1999; Meyer and Van de Peer 2005; Volff 2005). In addition, restriction-site associated DNA (RAD) sequencing based on next-generation sequencing (NGS) enables the rapid discovery of genome-wide genetic markers and high-throughput single nucleotide polymorphism (SNP) genotyping in mapping families and facilitates the construction of high-density genetic linkage maps in both model and non-model organisms (Baird *et al.* 2008; Davey and Blaxter 2010; Amores *et al.* 2011; Davey *et al.* 2011; Etter *et al.* 2011).

We have recently published a reference genome assembly (total genome length 779.4 Mb) of *O. melastigma* as a model species in environmental toxicology (Kim *et al.* 2018). In this study, we constructed a high-density genetic linkage map of *O. melastigma* using an F1 full-sib family. Using the genetic linkage map, sex QTL has been mapped in the genome of *O. melastigma* and the previous genome assembly was anchored onto the linkage map to improve the contiguity of the assembly. The development of a high-density genetic map is imperative to facilitate both genetic and genomic studies in *O. melastigma*. The present study will assist in a better understanding of genome-based research in molecular toxicology, genomics, CRISPR/Cas9, and epigenetics.

## Materials and methods

### Mapping cross

The marine medaka *O. melastigma* used in this study were obtained from the City University of Hong Kong (kindly provided by Dr. Doris W.T. Au) and maintained at Nagahama Institute of Bio-Science and Technology, Nagahama, Japan. A male and a female fish were bred to produce F1 progenies. In total, 58 F1 individuals were used to create a linkage group (LG).

### RAD sequencing

Genomic DNA was extracted from muscle tissue using DNeasy Blood & Tissue Kit (Qiagen, Hilden, Germany) according to the manufacturer’s instructions. The size and quality of DNA isolated was checked on 1% agarose gel by electrophoresis and the concentration was measured using Qubit florometer (Thermo Fisher Scientific, Waltham, MA, USA). Genomic DNA (40 ng) of each sample was digested with *Bgl*II (5 Unit) and *Eco*RI (5 Unit), ligated with a Y-shaped adaptor, amplified by polymerase chain reaction (PCR) with KAPA HiFi HS ReadyMix (Kapa Biosystems, Wilmington, MA, USA), and fragments were selected with E-Gel Size Select (Life Technologies, Carlsbad, CA, USA). The mean size of the selected fragments was 333 bp (CV 16.4%). Further details of the library preparation method are described in a previous study by Sakaguchi *et al.* (2015). RAD sequencing (RAD-seq) was performed using 58 F1 individuals and both parents with HiSeq2500 (Illumina, San Diego, CA, USA) with eight cycles for index read and 51 cycles for the reads of interest. For each parental sample, the same amounts were aliquoted in four different reaction tubes and sequencing of each reaction was carried out to reduce PCR amplification bias. All procedures related to RAD-seq including the library construction were performed by Clockmics Inc. (Osaka, Japan).

### Extracting RAD-tags and SNP genotyping by Stacks

Quality filtration of sequence reads was performed using Trimmomatic v.0.33 (Bolger *et al.* 2014) with parameter options of -0.33.jar SE -phred33 TOPHRED33 ILLUMINACLIP TruSeq3-PE-2.fa:2:30:10 LEADING:19 TRAILING:19 SLIDINGWINDOW:30:20 AVGQUAL:20 MINLEN:51. RAD-tag extraction and genotyping were performed with Stacks v.1.47 software (http://creskolab.uoregon.edu/stacks/) (Catchen *et al.* 2011). The sequence reads were aligned to the available reference genome **(GCF_002922805.1,** Kim *et al.* 2018**)** using GSnap (https://github.com/juliangehring/GMAP-GSNAP) with default parameters (-t 30 –n 1 –m 5 –i 2), which converted to BAM files. All RAD-tags catalog from the parental samples were extracted by Stacks using the *ref_map.pl* pipeline with the parameters –m10 and -P 3 and genotyping was called by the parameters of minimum number of 5 reads to call a homozygous genotype, a minimum minor allele frequency of 0.1 to call a heterozygote, and a maximum minor allele frequency of 0.05 to call a homozygote. Among RAD-tags, single nucleotide polymorphism (SNP) markers with maximum likelihood of 0 were selected for mapping and the SNP markers with genotypes of at least 53 F1 offsprings (> 90%) were collected for map construction using the command *genotypes* –r 53 of Stacks v.1.47. Data of raw sequences were deposited in the Sequence Read Archive (SRA) (http://www.ncbi.nlm.nih.gov/sra) under the accession number **PRJNA514812**.

### Linkage map construction

Linkage analysis of genetic markers (SNPs) was performed using JoinMap 5.0 (Wageningen, Netherlands, Van Ooijen 2018). The SNP markers with a significant segregation distortion (χ^2^ test, *P* < 0.01) were removed from the analysis of linkage map construction. Linkage groups were identified by the grouping parameters of independent Likelihood Odds Ratio (LOD) threshold of 5 provided in JoinMap 5.0. Map distances were calculated by the Kosambi’s mapping function and mapping algorithm used for building linkage map was regression mapping based on the default parameters. The regression mapping adds loci one by one starting from the most informative pair of loci. The best position of each added locus is searched by comparing the goodness-of-fit of the calculated map for each tested position. JoinMap performed three rounds of marker positioning with a jump threshold of 5 and we took second round of map as a final map as recommended by the manual. The linkage map was visualized using MapCart 2.32 (Voorrips 2002). The name of the linkage groups was matched with the homologous chromosomes of Japanese medaka.

### Anchoring the reference genome on to linkage map

Genetic markers in the linkage map were anchored to the reference genome **(GCF_002922805.1)** of the marine medaka *O. melastigma* using Chromonomer v. 1.08 (http://catchenlab.life.illinois.edu/chromonomer/). The integrated genome assembly based on the genetic map was re-assessed with benchmarking universal single-copy orthologs (BUSCO) v.3.0 (Simão *et al.* 2015) using the vertebrate database (OrthoDB v.9.0; https://www.orthodb.org/?page=filelist). The gene annotation of the final assembly was carried out using MAKER v.2.31.8 pipeline with manual curation (Suppl. Fig. S1) (Holt and Yandell 2011).

### Comparative analysis of two medaka genomes

The final linkage map based genome assembly of marine medaka was compared with the genome of Japanese medaka (Hd-rR strain) to compare the similarity between two medaka genomes using Mummer v.3.0 (http://mummer.sourceforge.net/manual/#coords).

### Sex linkage analysis

The mapping panel consisted of 27 males and 31 females. In order to examine sex determining mechanism in *O. melastigma* by linkage analysis, two completely linked sex markers (sex_xy and sex_zw) were additionally added in the genotype files. For sex_xy and sex_zw genotypes, male individuals were coded as heterozygotes and homozygotes, respectively, while female individuals were coded as homozygotes and heterozygotes, respectively. The linkage analysis of sex markers was performed with JoinMap 5.0 (Wageningen, Netherlands, Van Ooijen 2018). Since the sex trait is qualitative, the phenotype for sex was converted into binary code; male 1 and female 0. For QTL analysis, standard interval mapping was performed and significance was determined by permutation test (n = 1000) using MapQTL 6.0 (van Ooijen *et al.* 2002).

### Data availability

Sequenced species *O. melastigma* is available upon request. Suppl. Data contains all supplemental tables and figures. Suppl. File S1 contains the names of 2481 SNP, genotype, map positions, and tag sequences in Map info tab and phenotypes for sex of all individuals were in Phenotype tab. Suppl. File S2 contains the re-scaffolding process and results by Chromonomer. Suppl. File S3 includes the gene list annotated in the sex-determining region. Raw data of RAD seq were deposited to GenBank under the accession number **PRJNA514812**. The genome sequence data are available in GenBank with accession number **PRJNA401159**. The genome JBrowse is available at http://rotifer.skku.edu:8080/Om2. Supplemental material available at FigShare: https://doi.org/10.25387/g3.7811978.

## Results

### Constructing genetic linkage map of the marine medaka Oryzias melastigma

**Figure 1:**
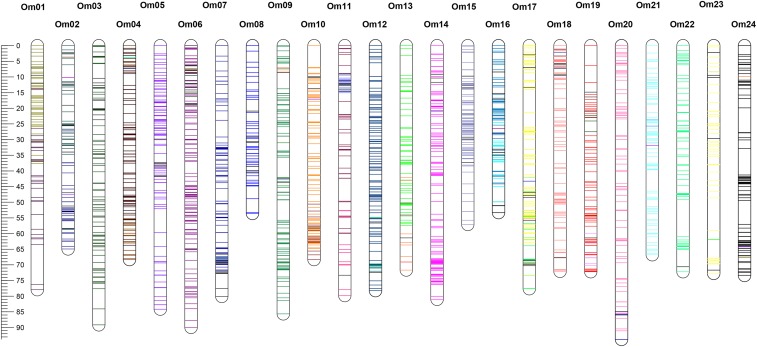
A linkage map of the marine medaka *Oryzias melastigma*. The map consists of 24 linkage groups and the bars on each linkage group represents single nucleotide polymorphism (SNP) markers. Colors of bars indicate the reference-assisted chromosome assembly scaffolds that SNP was extracted. (Name, sequences, and position of SNP are included in the Suppl. File 1.)

Stacks software extracted 113,367 RAD-tags from *O. melastigma* genome. Among them, the number of putative SNP markers was 34,040. To distinguish polymorphisms from sequencing errors, we collected 24,441 SNP markers with Likelihood Ratio of 0, including 8,518 SNP markers that have been located in the same RAD-tags more than twice. Among the remaining 15,922 loci, 4,497 SNP loci were successfully genotyped in at least 53 F1 individuals (>90%). After removing the markers that showed a segregation distortion (*P* < 0.01), 3,732 were finally used for building a genetic map. The 3,730 markers were grouped into 24 LGs with a LOD ≥ 5.0 by independence LOD and two remaining markers were not linked with any of those groups. The regression mapping function by JoinMap positioned 2,481 SNPs in the second rounds of mapping by comparing the goodness-of-fit (Suppl. File S1). The 24 LGs were consistent with the number of chromosomes (n = 24) in *O. melastigma* (Uwa *et al.* 1983). The total map length was 1,784 cM and each LG included 57-173 markers with an average marker interval of 0.72 cM (Figure 1 and Table 1).

**Table 1:** Summary of the genetic linkage map of *Oryzias melastigma*

LG	Group ID	No. markers mapped	Map Length (cM)	*Oryzias latipes* Chromosome
Om01	Group 7	91	77.92	1
Om02	Group 21	98	64.935	2
Om03	Group 5	107	89.184	3
Om04	Group 6	108	68.313	4
Om05	Group 13	102	84.132	5
Om06	Group 4	173	89.972	6
Om07	Group 12	118	80.081	7
Om08	Group 24	57	53.584	8
Om09	Group 17	111	85.671	9
Om10	Group 16	117	68.297	10
Om11	Group 1	103	79.839	11
Om12	Group 8	123	78.25	12
Om13	Group 15	74	71.657	13
Om14	Group 14	142	81.03	14
Om15	Group 18	106	57.118	15
Om16	Group 19	88	53.324	16
Om17	Group 3	128	77.589	17
Om18	Group 2	117	72.043	18
Om19	Group 22	99	72.178	19
Om20	Group 23	83	93.802	20
Om21	Group 20	93	66.785	21
Om22	Group 9	79	72.197	22
Om23	Group 11	71	72.7	23
Om24	Group 10	93	73.366	24
Total		2481	1783.967	

### Re-scaffolding of the reference genome with the genetic map

Using the SNP marker information in the high-density genetic linkage map, 810 markers were anchored to 134 scaffolds and among them, 35 were split into 1 to 9 positions, producing a total of 260 integrated scaffolds (Tables 2, Suppl. Table S2, and Suppl. Fig. S2). After integration, the length of the genome scaffolds aligned on the map was 712,537,413 bp (Table 2). Out of the 260 integrated scaffolds, the orientation was determined in 160 scaffolds spanning 670,530,120 bp, which accounted for 94% of the total scaffold length in the linkage map (Table 2). Among 40 reference-assisted chromosome assembly (RACA) scaffolds previously published (Kim *et al.* 2018), 20 RACA scaffolds (RACA3, 5, 6, 7, 8, 9, 11, 12, 14, 15, 18, 21, 22, 23, 25, 26, 27, 29, 30, 40) were aligned to the 13 linkage groups (Om23, Om21, Om20, Om19, Om17, Om16, Om14, Om 12, Om11, Om10, Om09, Om08, Om01) without any modification (Suppl. Table S1). Other RACA scaffolds showed the major alignment of one linkage group with the partial alignment of another linkage groups (Suppl. Table S1). Among them, RACA 31 showed the most frequent rearrangement during the anchoring process, which was a major alignment with Om 7 and partial alignments with another 4 linkage groups (Om20, Om18, Om17, and Om02) (Suppl. Table S1). Four linkage groups (Om03, Om05, Om15, and Om22) were completely aligned by each of 4 RACA scaffolds (RACA4, RACA16, RACA33, and RACA36) respectively (Table 2), although some parts of sequences aligned in the linkage groups were inserted from small scaffolds (Suppl. File S2) and the parts of RACA scaffolds were located in parts of another linkage groups (Supp. Table S1). Overall, the final genetic map based genome assembly consisted of 8,493 scaffolds; 24 linkage map-based scaffolds (90.7%) and 8,469 unanchored scaffolds (9.3%). The total genome length was 779 Mb with an N50 value of 29,978,720 bp (Table 3). BUSCO analysis indicated that the final genome assembly of *O. melastigma* represented 96.8% of the complete copy in the vertebrate gene model (Table 4). The genome annotation pipeline in the final assembly was defined as 24,507 genes (http://rotifer.skku.edu:8080/Om2), ranging from 661 to 1,216 genes per LG (Table 3 and Suppl. Table S3).

**Table 2:** Physical lengths of linkage map anchored with the reference genome assembly in *Oryzias melastigma*^#^

LG	Physical Length (bp)	No. of anchors	No. of scaffolds	No. of oriented scaffolds	Length of Oriented scaffolds (bp)	RACA scaffolds anchored to linkage groups
Om01	32,649,675	37	8	6	31,960,121	1, 33, **39, 40**
Om02	22,231,404	37	22	11	18,905,508	17, 21, 31,33, 35, **37, 38**
Om03	35,272,651	34	11	7	33,734,277	**36**
Om04	31,689,622	26	10	8	31,276,484	4, **34, 35,** 38
Om05	37,216,997	27	11	8	36,448,571	**33**
Om06	35,155,257	50	14	11	34,854,582	**32**, 35, 36
Om07	33,183,412	35	13	8	32,581,825	13, **31,** 37
Om08	25,074,844	16	9	5	24,678,101	1, 2, **30**
Om09	33,864,506	34	15	8	28,264,709	19, **28, 29**, 34, 37
Om10	29,394,598	44	13	5	21,895,787	17, **26, 27,** 33
Om11	27,364,941	33	13	9	26,207,853	**23, 24, 25,** 36, 39
Om12	27,293,507	48	9	6	26,239,884	**21, 22**
Om13	33,844,731	23	10	5	31,595,510	10, **19, 20**, 24, 32
Om14	29,986,920	41	9	6	27,349,872	**17, 18,**
Om15	31,267,159	23	9	7	30,446,100	**16**
Om16	31,579,375	28	9	6	27,524,063	**14, 15,** 28, 32, 38
Om17	34,593,980	36	22	13	33,270,277	10, **11, 12, 13**, 16, 31
Om18	25,749,489	50	16	7	23,208,882	**10,** 15, 31
Om19	24,827,326	39	11	8	23,944,248	**8, 9,** 20, 36
Om20	26,018,873	35	4	2	23,810,479	**7**, 31
Om21	28,918,309	30	3	3	28,918,309	**5, 6**
Om22	27,453,564	28	3	2	27,180,060	**4**
Om23	23,210,987	33	9	5	22,600,831	**2, 3,**
Om24	23,210,987	23	7	4	23,633,787	**1**, 39
Total	712,537,413	810	260	160	670,530,120	

# Bold numbers were mainly anchored scaffolds on the linkage map.

**Table 3:** Statistics of the final genome assembly before and after anchoring in *Oryzias melastigma*

	Reference genomes	
Statistics	RACA	Linkage map based
Number of scaffolds	8,602	8,493
Length of scaffolds (bp)	779,456,607	778,703,520
N50 (bp)	23,737,187	29,987,720
Largest scaffold (bp)	37,948,421	37,217,997
Gap (%)	5.29	5.2
GC content (%)	39.04	37.02
Number of unanchored scaffolds		8,469
Length of unanchored scaffolds (bp)		66,142,507
Number of genes	23,528	24,506
Total genes length (bp)	51,834,196	24,784,506
Average genes length (bp)	2,586	1,011
Maximum gene length (bp)	80,775	26,364
GC content (%)	54.34	54.27

**Table 4:** Assessment of LG-based assembly completeness

Vertebrata DB	%	No. of genes (n = 2586)
Complete BUSCOs (C)	96.8	2504
Complete and single-copy BUSCOs (S)	95.8	2477
Complete and duplicated BUSCOs (D)	1	27
Fragmented BUSCOs (F)	1.2	32
Missing BUSCOs (M)	2	50

### Comparative genomic analysis of two medaka genomes

**Figure 2:**
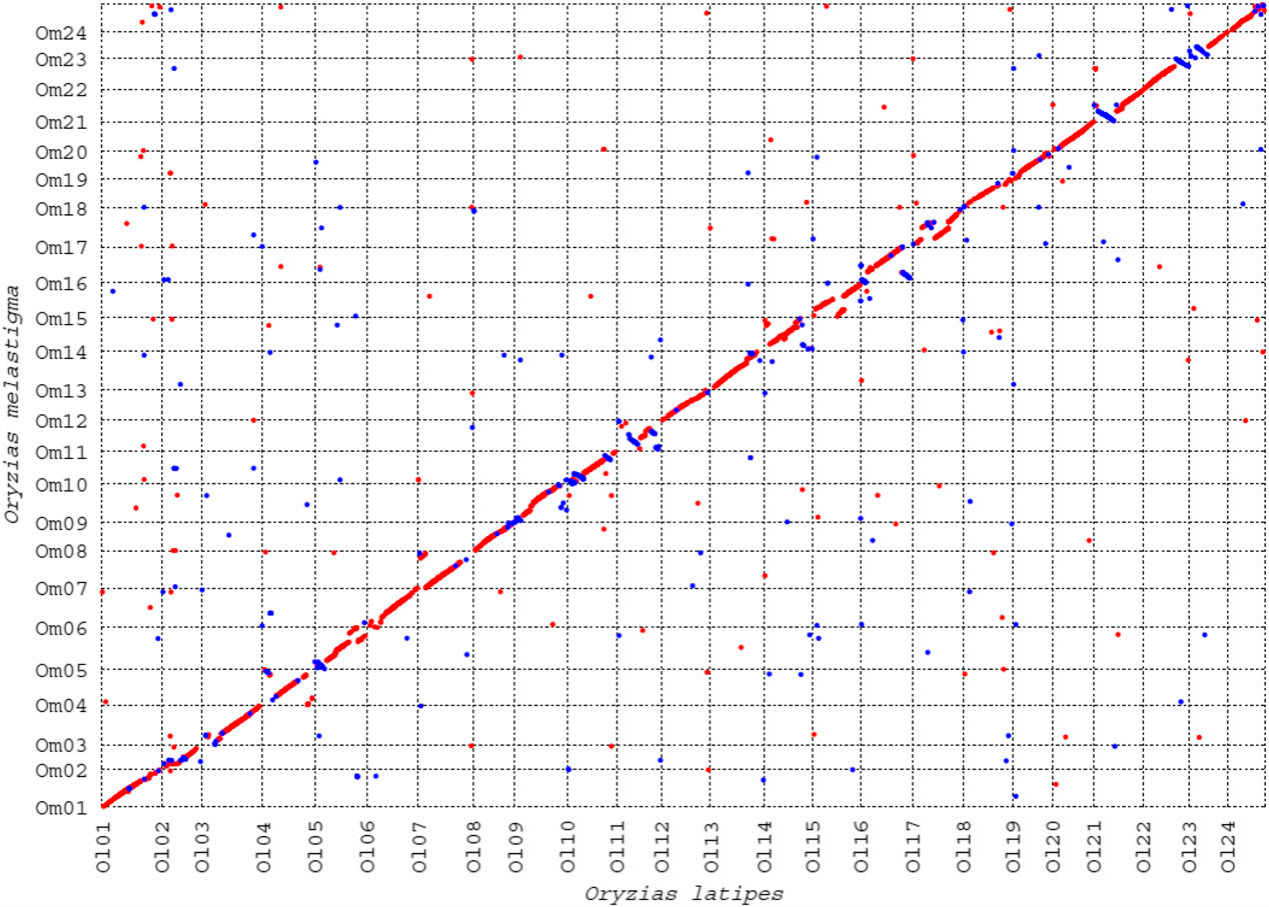
Genome-wide comparison of the genomic sequences between *Oryzias melastigma* and *Oryzias latipes* using Mummer. Red and blue dots represent forward and reverse match, respectively.

The final genome assembly integrated with a genetic map provides an efficient resource for comparative genomic analysis with other medaka genomes such as a Japanese medaka (*O. latipes*). The 24 genetic map-based scaffolds showed good homology in gene contents and sequences similarity with chromosomes in *O. latipes*, Most LG-based scaffolds of marine medaka showed collinear relationships completely or in a majority with the counterpart chromosomes of *O. latipes* (Figure 2 and Suppl. Fig. S3). Other LG/chromosomes in *O. melastigma* showed disrupted collinearity due to intrachromosomal rearrangements or possible errors in linkage map. Reversely matched parts in the collinearly related scaffolds were most likely caused by the undetermined orientation of scaffolds involved in the anchoring process. In addition, some LG-based scaffolds showed in/del regions (Om05, Om08, Om11, Om14, Om15, Om16, and Om17) compared to Japanese medaka chromosomes.

### Mapping of sex-determining regions

The linkage analysis of two sex markers (sex_xy and sex_zw) showed that both markers were more or less linked to markers in the Om10. Sex_xy showed the highest LOD scores with 17018 (LOD = 17.06) and strong linkages with other markers in this linkage group (Suppl. Table S4). Although sex_zw showed the highest LOD value (7.42) with 16660, which also had the same LOD with sex_xy, the LOD scores of sex_zw with other markers indicated weak or suspect linkage (Suppl. Table S4). This investigation suggested that the *O. melastigma* has XY sex-determining system.

**Figure 3:**
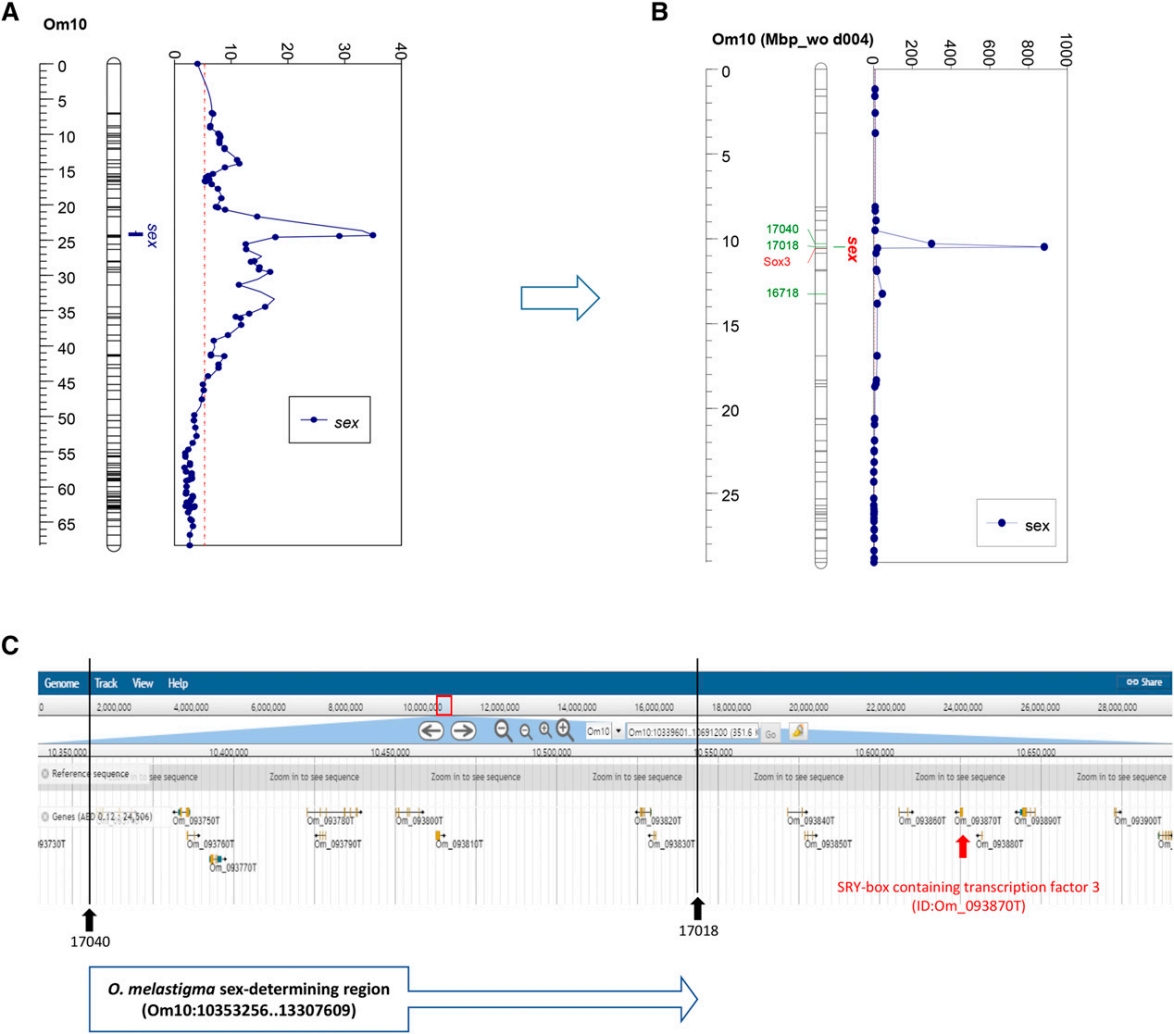
Quantitative trait locus mapping for sex traits on the Om10 scaffold of *Oryzias melastigma*. (A) Standard interval mapping. Scales on the left represents the map position (cM) of linkage group Om10; Scales on the top of the graph represents the value of the Likelihood Odds Ratio (LOD) scores. The red dotted line indicates the threshold of significance (LOD = 5.3). (B) Standard interval mapping without d004 individual. Scales on the left represents the physical (Mbp) of Om10 scaffold; Scales on the top of the graph represents the value of the LOD scores. The red dotted line indicates the threshold of significance (LOD = 5.3). (C) Gene lists in the sex-determining region of Om10 scaffold.

A significant QTL for sex was detected in Om10 with the LOD significance threshold of 5.3 based on the permutation test (Figure 3A). Among 2,481 markers mapped in the Om linkage groups, 58 markers showed the significant association with sex (Suppl. Table S5), most of which were markers from the RACA27. The most significant two markers associated with sex were 17040 (24.3 cM) and 17018 (24.4 cM) with LOD of 35 (Suppl. Table S5). The genotypes of those markers were completely linked with the sex phenotype of all individuals, except for one animal (d004), which could be caused by wrong phenotyping or sex-reversal. When the animal was removed from the QTL analysis, LOD scores of 17018 and 17040 increased to 881.12 and 299.4, respectively (Figure 3B). Physical location of sex-determining region was mapped between 10,353,256 bp and 10,538,878 bp in Om10, spanning approximately 186 kb, and 10 genes were located in the regions without any obvious candidate gene (Figure 3C). However, the sox3 was located on the 10,625,885 bp, which was 87 kb apart from the mapped region (Suppl. Table S3; Figure 3C).

## Discussion

A high-resolution genetic map is very useful in diverse genomic research and has been applied to many fish species (Amores *et al.* 2011; Li *et al.* 2015; Shao *et al.* 2015; Xiao *et al.* 2015; Wang *et al.* 2015; Kanamori *et al.* 2016). In this study, a high-density genetic map was constructed in *O. melastigma* using RAD sequencing and was used for verifying the previously published marine medaka reference genome and for aligning the scaffolds at the chromosomal level. The genetic map of *O. melastigma* consists of 24 LGs with 2,481 SNP markers, which cover 24 chromosomes (Uwa *et al.* 1983). The 810 genetic markers were anchored to the genetic map (Table 2 and Suppl. Figure 2), which mapped 90.7% (713 Mb) of the reference genome assembly sequence onto the genetic map (Table 3). Although all SNP markers were extracted by the alignment of RAD sequences against the reference genome of marine medaka (Kim *et al.* 2018), the number of anchored markers were lower than anticipated. Many markers in the LGs were excluded from the integrating process due to inconsistency between the genetic map and reference assembly, indicating possible errors in marker order and/or *de novo* assembly. Indeed, in most cases the markers tended to be highly concentrated in the narrow regions, suggesting that more recombinant individuals will be required to obtain a more precise marker order. Also, it is likely that the size of the mapping family was not big enough for the marker concentrated regions, compared with the number of markers analyzed, resulting in relatively low number of anchored markers. Despite this shortage, the marine medaka genetic map was still integrated with the reference assembly, considering other fish species had a similar or slightly lower level of scaffolds mapping onto the genetic map (Tine *et al.* 2014; Li *et al.* 2015; Wang *et al.* 2017).

Previously, we have developed a reference genome for the marine medaka *O. melastigma* in several steps (Kim *et al.* 2018). *De novo* assembly was performed using the combination of Platanus and Haplomerger2 assemblers (Kajitani *et al.* 2014; Huang *et al.* 2017). The contiguity of *de novo* genome assembly was further increased by RACA (Kim *et al.* 2013), which assisted in the construction of highly ordered and oriented scaffolds of *de novo* assembly and reassembled scaffolds into longer chromosomal fragments using the comparative genome information of closely related species. RACA assembly of *O. melastigma* generated 40 gigantic scaffolds (Suppl. Table 1), which account for 674 Mb (86.5%) of the total genome sequence (Kim *et al.* 2018). Taken together, the genetic map-integrated assembly improved the reference sequences by mapping 90.7% of genome sequences onto 24 LGs with 94% determined orientation of mapped scaffolds (Table 2). The BUSCO and N50 demonstrated a better quality of integrated final genome assembly with values increased to 96.8% and 29,987,720 bp, respectively (Tables 3 and 4).

Quantitative trait locus (QTL) analysis identified that Om10 was strongly associated with sex in *O. melastigma* and two markers, 17040 and 10718, showed the most significant association for sex, without any recombinant between genotype and phenotype. LOD scores of 17040 (LOD = 34.99) is little higher than that of 10718 (LOD = 34.98), which is likely associated with the missing genotype in one individual for 10718 (Suppl. Table S5). Thus, sex determining locus should be located between 17018 and 17040. Although no obvious candidate gene for sex was found in the mapped sex-determining regions, we noticed *sox3* outside the mapped region (Figure 3C). It was intriguing because a *cis*-regulatory element in the downstream region of *sox3* on Y chromosome plays a key signal to sex determination in *O*. *dancena* (Takehana *et al.* 2014), suggesting that the same *cis*-regulatory element might be present in the mapped regions of *O. melastigma*. For further analysis, fine mapping with additional recombinants and the functional analysis of candidate genes or regulatory elements need to be performed in the mapped sex-determining region.

In summary, a high-density genetic map is a very useful resource to determine the accuracy of *de novo* genome assembly, especially with massively parallel short sequencing reads. In addition, the integration of a genetic map with reference genomes will be a useful resource in various genomic studies including comparative genomic analyses, fine mapping of QTL, positional cloning of candidate genes, and CRISPR/Cas9 studies.
